# Polyelectrolyte Complex Coating for Mitigating Decomposition at Argyrodite and Conductive Carbon Interfaces in Solid‐State Batteries

**DOI:** 10.1002/cssc.202502431

**Published:** 2026-04-18

**Authors:** Sudeshna Sen, Bing‐Xuan Shi, Nina Herrmann, Felix Schnaubelt, Felix Walther, Joachim Sann, Felix H. Richter

**Affiliations:** ^1^ Institute of Physical Chemistry Justus‐Liebig‐University Giessen Giessen Germany; ^2^ Center for Materials Research Justus‐Liebig‐University Giessen Giessen Germany; ^3^ VSPC Pty Ltd Brisbane Queensland Australia; ^4^ ZEISS Semiconductor Manufacturing Technology Oberkochen Germany

**Keywords:** interface degradation, polyelectrolyte complex, polymer coating, solid electrolyte, solid‐state batteries

## Abstract

Sulfide‐based solid electrolyte batteries (SEBs), which are an important type of solid‐state battery, show strong potential for commercializing solid‐state battery technology in large scale with high energy density. For delivering high capacity, maximum utilization of cathode active materials is a prime criterion, which can be attained using carbon additives to ensure electronic connectivity of all cathode particles. Fibrous carbon additives such as vapor‐grown carbon fibers (VGCFs) are often preferred in SEBs. However, degradation of sulfide‐based solid electrolytes such as Li_6_PS_5_Cl (LPSCl) at the interfaces with cathode active material and VGCF lowers cell capacity. Coating of carbon surfaces is a viable method to mitigate electrolyte decomposition. Here, we report a new polyelectrolyte‐based coating on VGCFs as protective interlayer for LiIn|LPSCl|LPSCl‐NCM‐VGCF (^LiIn^SEB^NCM^) cells. We use cyclic voltammetry to evaluate oxidation of electrolyte at the VGCF interface along with galvanostatic charge–discharge cycling. The polymer coating decreases argyrodite oxidation at the VGCF|LPSCl interface and improves cycling capacity. An interplay between coating thickness and aggregation of VGCF fibers is observed, which leads to an optimum of polymer coating layers to maximize cycling performance.

## Graphical abstract

Sulfide‐based solid‐state batteries can achieve high capacity using carbon additives like vapor grown carbon fibers (VGCF), but interfacial degradation with sulfide electrolytes reduces performance. A new polyelectrolyte complex coating on VGCF mitigates electrolyte oxidation and enhances cell cycling stability by increasing VGCF‐solid electrolyte interface stability.

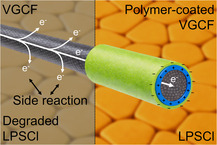

## 1 Introduction

Solid‐state batteries, of which sulfide‐based solid electrolyte batteries (SEBs) are a promising representative, have gained strong interest due to high projected energy density and safety [1]. SEBs that use sulfide‐based solid electrolytes (SEs) have the potential to achieve the demand for high power and energy density [2]. Sulfide‐based SEs have high ionic conductivity of up to 32 mS·cm^−1^ for Li_9.54_(Si_0.6_Ge_0.4_)_1.74_P_1.44_S_11.1_Br_0.3_O_0.6_ [3], 10 mS·cm^−1^ for Li_10_GeP_2_S_12_, and 2 mS·cm^−1^ for Li_6_PS_5_Cl (LPSCl) [4, 5, 6]. LPSCl in particular is a widely used and commercially available SE. The conductivity of argyrodite‐type SEs can be improved up to 25 mS·cm^−1^ with suitable cation doping and anion site tuning. Few examples of argyrodite family showing ionic conductivity >2 mS·cm^−1^ are Li_6.5_Sb_0.5_Ge_0.5_S_5_I (16.1 mS·cm^−1^) [7], Li_5.5_AsS_4.5_Br_1.5_ (15.4 mS·cm^−1^) [8], Li_9.54_Si_1.74_P_1.44_S_11.7_Cl_0.3_ (25 mS·cm^−1^) [9], Li_5.5_PS_4.5_Cl_1.5_ (9.4 mS·cm^−1^) [10], and Li_5.7_PS_4.7_Cl_1.3_ (6.4 mS·cm^−1^) [11].

The mechanical properties of sulfide‐based SEs allow to prepare suitable battery interfaces at room temperature by simple pressure‐based dry or slurry‐based processing techniques [12]. Overall, the properties of LPSCl comprise ease of processing, fair electrochemical stability, and reasonable ionic conductivity [13]. High‐nickel cathodes, such as LiNi_*x*_Mn_*y*_Co_1−*x*−*y*_O_2_ (NCM), provide high energy density and cost efficiency [14, 15]. Together, this combination of LPSCl and NCM is of interest for both industrial applications and academic research.

Still, several challenges remain. Poor cyclability of LPSCl‐based SEBs is caused by electrochemical degradation at interfaces. These include volume expansion, electrolyte degradation, contact loss at interfaces, and particle fracture [16]. The rate capability of SEBs depends on electrode composition and type of carbon additives [17, 18], such as vapor‐grown carbon fibers (VGCFs), carbon nanotubes (CNTs), and carbon black [19]. Randau et al. reported the performance of different carbon additives for Li_3_PS_4_‐based SEBs: The first discharge capacity is highest for VGCF and in sequence of VGCF > Super C65 > no carbon additive >  CNTs > Ketjenblack EC600JD (KBC) [19]. Even though the impact may vary between different SEs, the use of a carbon additive generally causes more electrolyte decomposition and capacity loss than using no carbon additives [17, 19]. This is because fibrous carbon enhances electronic pathways [15], but increased SE‐VGCF interfacial degradation leads to stronger degradation and capacity loss [16].

Interfacial degradation in SEBs occurs primarily at the LPSCl/NCM, current collector/LPSCl, and VGCF‐LPSCl interfaces. Oxidation of LPSCl above 1.7 V versus In/LiIn (2.3 V vs. Li^+^/Li) generates elemental sulfur and P−[S]_*n*_−P species at these current‐carrying interfaces in the first cycle [20, 21]. Subsequently, at a state of charge (SOC) around 4.2 V versus Li^+^/Li, oxygenated reactions at LPSCl/NCM interface lead to the formation of sulfate and phosphate passivation layers and the rock salt phase on NCM, further degrading the interface [22, 23, 24]. Moreover, polysulfides can be oxidized to sulfide (S^2−^) or sulfite (SO_3_^2−^) through reactions with residual lithium compounds such as Li_2_O [25, 26]. These reaction products hinder ion transport and charge transfer, compromising the initial Coulomb efficiency (CE) [5, 20]. However, P−[S]_*n*_−P formation is the main decomposition process at current‐carrying interfaces, driven by VGCF, highlighting the issue of VGCF on interfacial degradation [16]. Surface coatings on VGCF are an effective way to decrease LPSCl‐VGCF interfacial degradation. Some inorganic materials have been developed as coatings for VGCF, such as Al_2_O_3_ [17] and ZnO [27]. However, inorganic coatings can fracture or detach, making it difficult for them to adapt to changes in cathode volume [28]. Polymers have a lower elastic modulus (∼6 GPa) than thiophosphate‐based SEs (∼20 GPa) [28, 29], allowing them to better accommodate cathode volume changes. For instance, electronically conductive polymer (PEDOT:PSS) has been coated on VGCF using molecular layer deposition [30]. However, electronically conductive polymer can also lead to LPSCl‐VGCF interfacial degradation. Therefore, applying an electronically insulating coating with precise control over coverage and thickness is important.

Nanometer‐scale tuning of coatings is possible with atomic layer deposition (ALD) and molecular layer deposition (MLD)X‐ [17, 30]. However, these methods are costly and hard to scale for mass production. Polymer coating applied by wet coating from solution offers a simpler alternative to inorganic coatings using deposition methods, with significant advantages in terms of lower cost, reduced hazards, and structural versatility. Shi et al. reported 2‐ to 4‐nm‐thick polyelectrolyte‐based coatings on NCM to stabilize LPSCl‐based SEB interfaces [31, 32]. The polyelectrolyte ionic functional group binds to inorganic surfaces through electrostatic interactions during surface coating. The efficient binding ability of the polymer coating mitigates contact loss between cathode particles and decreases oxidation of LPSCl at the LPSCl‐NCM interface. As a result, better cyclability and rate capability are observed for the polyelectrolyte coatings.

In the present study, we propose a new polyelectrolyte complex coating for VGCF, using positively charged polymer poly(diallyldimethylammonium chloride) (PDADMACl) and negatively charged polymer poly(sodium 4‐styrenesulfonate) (PSS). The polymer chosen is not electronically conducting and is a suitable choice as an electronically insulating protective layer. The electrostatic interaction between polycation and polyanion backbones create an insoluble coating on the VGCF surface [33, 34]. Fine‐tuning of polymer morphology at the VGCF interface is achieved, giving flexibility in polymer layer thickness and structure.

## 2 Experimental Section

### 2.1 Preparation of Polymer‐Coated Vapor‐Grown Carbon Fiber

To prepare VGCF fibers for coating, they underwent an acid treatment by stirring in conc. HNO_3_ at 70°C for 3 h. Afterward, the fibers were filtrated and washed until the pH was neutral. The fibers were then dried in a vacuum oven at 50°C. Subsequently, the layer‐by‐layer (LBL) approach consists of multiple solution‐based coating steps using the positively charged polymer poly(diallyldimethylammonium chloride) (PDADMACl) and the negatively charged polymer poly(sodium 4‐styrenesulfonate) (PSS) in an alternating manner. First, a coating of the positively charged PDADMACl was applied onto the fibers and then one of the negatively charged PSS. These two steps constitute one coating cycle. This coating cycle was repeated twice. For each coating step, the respective polymer was dissolved in a 0.5 M NaCl solution. The weight ratio of carbon fibers to polymer was 1:1. The carbon fibers were added to the solution, and the resulting dispersion was ultrasonicated for 30 min at 80 Hz. Afterward, the dispersion was stirred at room temperature for 1.5 h. The fibers were then filtered and dried in a vacuum oven at 50°C for 18 h. The preparation of L2@VGCF was slightly modified to enhance purity. This sample was washed by centrifugation after each coating cycle to reduce excess polymer residue.

### 2.2 Zeta‐Potential Measurements

The surface chemistry and change of the surface charge of the fibers during the polymer coating are observed by measuring the zeta‐potential of the carbon fibers before and after polymer coating. For the measurement, the fibers were dispersed in distilled water by ultrasonication for 10 min. The measurement was performed using a Zetasizer NanoZS by Malvern Instruments at room temperature. The measurement cell DTS0012 made of polystyrene was filled with the dispersion to the maximum fill line. Three measurements were done, and the average zeta‐potential was determined including the deviation.

### 2.3 Scanning Electron Microscope Measurements

Powder samples were mounted onto a sticky carbon tape, and loose particles were removed with compressed air. Images were recorded with a Merlin scanning electron microscope (SEM) by Zeiss Instruments. A current of up to 3 nA and a voltage of 5 kV were applied. Images were recorded in a magnification range of 500–30,000 with a secondary electron as well as an in‐lens detector. The distance between the sample and detector was between 5 and 7 mm. The recorded images were analyzed using the software ImageJ. Therefore, 100 fiber diameters were measured with the software ImageJ for each sample, and the average diameter and deviation were calculated and noted in Table S1. For cross‐section SEM of cathode composites, the battery was disassembled first. The cathode composite pellet was recovered and mounted on a sample holder with sticky carbon tape. The sample was transferred using a Leica shuttle system to avoid exposure to air.

### 2.4 Thermogravimetric Analysis Characterization

Thermogravimetric analysis (TGA) coupled with mass spectrometry (MS) was performed to determine the polymer content in samples. The analysis was carried out using a Netzsch STA 409 simultaneous thermal analyzer in a temperature range from 30°C to 1000°C in synthetic air (at a heating rate of 10 K min^−1^). The VGCF, L1@VGCF, and L6@VGCF samples were recorded at 10 K min^−1^. For the L2@VGCF, the data was recorded at 5 K min^−1^. All data was recorded in synthetic air. The coupled mass spectrometer QMS 403 Aëolos (Netzsch) measured mass to charge ratios between 12 and 100. The signals corresponding to CO_2_, H_2_O, SO_2_, NO_2_, and NO were monitored during thermal analysis.

### 2.5 X‐Ray Photoelectron Spectroscopy

X‐ray photoelectron spectroscopy (XPS) was conducted with a PHI5000 Versa Probe II from Physical Electronics Inc. VGCF powders were compacted into small cylindrical Teflon vessels (diameter 3 mm) and secured with tape onto the sample holder. For ex‐situ analysis of the cycled cathode, a piece of the cell stack was directly placed onto the sample holder and fixed with adhesive tape. Sample transfer from the glovebox to the measurement device was conducted under inert conditions. Monochromatic Al Kα radiation (1486.6 eV) with a source power of 50 W and a voltage of 15 kV was used. The analysis beam diameter was 200 μm and a pass energy of 43 eV was applied. For weak signals, e.g. nitrogen, the pass energy was increased to 95 eV. A dual beam charge neutralization (ion beam combined with a low‐energy electron beam) was utilized to mitigate surface charging.

To clean the surface and to enable a relatively soft material abrasion for depth profiling, depth profiling was performed with an Ar^+^ ion sputter gun set to an acceleration voltage of 0.5 kV. The sputtered area is used as 2 × 2 mm^2^.

Data analysis was carried out with Casa XPS. The spectra were calibrated to the C1s signal of adventitious carbon at BE = 284.8 eV at first, followed by a second calibration to the S2p signal of the PS_4_^3−^ unit (BE: 161.6 eV). Shirley background and GL (30) line shapes were used for data analysis.

### 2.6 Electrochemical Measurements

All electrochemical experiments were done using a VMP‐300 Biologic potentiostat and were carried out in a temperature‐controlled chamber at 25°C. All cells were assembled inside an argon‐filled glovebox (LabMaster, MBraun, Garching, Germany, <0.1 ppm of O_2_, <0.1 ppm of H_2_O). An in‐house‐made cell casing with an inner diameter of 1 cm was used.

#### 2.6.1 Assembly for Cyclic Voltammetry

For cyclic voltammetry (CV), InLi|LPSCl|LPSCl‐VGCF cells were prepared in a press cell setup. As separator, 80 mg of LPSCl was filled into the press cell and manually compressed. On the separator, 30 mg of the prepared composite was added. The powder stack was compressed uniaxially at 3T pressure for 3 min. Afterward, the anode consisting of an indium (9 mm diameter) and a lithium disc (4 mm diameter) was added onto the other side of the separator. After cell assembly, the cells were placed in an external frame with an axial stack pressure of 40 MPa.

#### 2.6.2 Cyclic Voltammetry

Before the CV measurement, the cells were rested at open circuit voltage (OCV) for 3 h. CV was performed using a two‐electrode setup (counter electrode functions as reference electrode). From the OCV, a voltage sweep to 4 V versus the reference electrode was performed with a scan speed of 0.1 mV·s^−1^, from where the sweep was reversed until 0 V. For the more detailed analysis, CV for LiIn|LPSCl|L*n*@VGCF‐LPSCl (*n* = 0; 2) recorded at 0.05 mV·s^−1^ scan rate with narrow voltage range of 2.2–4.2 V at first and second cycles. This measurement avoids the reduction of electrolyte. No effect of reductive decomposition product could influence the study of electrolyte oxidation segment only. For investigation of oxidation of electrolyte and polymer decomposition further, similar cell is also scanned at in stepwise manner between 2.6 and 3.4 V with interval of 0.4 V at 0.05 mV·s^−1^ scan rate.

#### 2.6.3 Battery Assembly and Cycling

Cell cycling was carried out with InLi|LPSCl|LPSCl‐NCM‐VGCF cells. Firstly, 80 mg of argyrodite was added into the cell and slightly compressed to form the separator. To prepare the composite cathode, 12 mg of NCM, 9 mg of premortared LPSCl, and 0.3 mg of carbon fibers were mixed using an agate mortar and pestle for 15 min. About 12 mg of the prepared composite was added on top of the LPSCl separator. After compressing uniaxially, the anode was assembled on the other side of the separator by adding an indium disc (9 mm diameter) and a lithium disc (4 mm diameter). Cells are cycled under stack pressure of 40 MPa. Cells were charged until the upper cutoff potential of 3.6 V and discharged to the lower cutoff potential of 2.0 V versus In/InLi. Cells were cycled up to 60 cycles at 0.1C rate (1C corresponds to 200 mA·g^−1^). The weight of cathode active material is 8.3 mg.

#### 2.6.4 Electrochemical Impedance Spectroscopy

Electrochemical impedance spectroscopy (EIS) measurements were performed using a VMP‐300 Biologic potentiostat. EIS data were collected during the first 10 cycles of the cycling test. Prior to impedance measurements, the cells were charged at a current density of 0.21 mA·cm^−2^ (0.1C) to a cutoff potential of 3.15 V vs. In/LiIn. The potential was then held at 3.15 V vs. In/LiIn until the current decreased below 0.042 mA·cm^−2^. Subsequently, EIS measurements were conducted over a frequency range from 1 MHz to 100 µHz using variable AC perturbation amplitudes. An amplitude of 10 mV was applied from 1 MHz to 10 mHz with 10 points per decade, 5 mV from 10 mHz to 1 mHz with 8 points per decade, and 3 mV from 1 mHz to 100 µHz with 8 points per decade. The amplitude was adjusted as a function of frequency to maintain a near‐linear current response and minimize measurement errors, as lower AC amplitudes improve linearity in impedance measurements.

## 3 Results and Discussion

**FIGURE 1:**
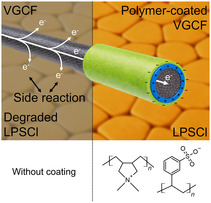
Schematic representation of the polyelectrolyte complex coating on VGCF. The structures of constituent polymers are also shown for poly(diallyldimethylammonium chloride) (PDADMAC) and poly(sodium 4‐styrenesulfonate) (PSS).

VGCF is coated with polycation (PDADMAC) and polyanion (PSS) in alternating LBL manner from Layer 1 (L1@VGCF) to Layer 6 (L6@VGCF), starting with the polycation, resulting in a polyelectrolyte complex coating on VGCF. The influence of polymer coatings for VGCF on the degradation in LPSCl‐based composites was tested (Figure 1). Each cycle of coating consists of one polycation and one polyanion coating step (Coating Cycle 1: Layer 1 PDADMAC and Layer 2 PSS). The presence of the polymer layer on VGCF is characterized by a switch of zeta‐potential of the coated VGCF samples. Each polycation coating step results in a positive zeta‐potential (L1@VGCF: +39.7 ± 5.2 mV) and each polyanion coating produces samples with a negative zeta‐potential (L1@VGCF: −51.5 ± 6.4 mV). The surface charge switches periodically between positive and negative after each layer application, which confirms the successful deposition of a new coating layer, see the zeta‐potential measurements shown in Table 1.

**TABLE 1:** Zeta‐potentials of VGCF and polymer‐coated L*n*@VGCF measured after each step of the LBL coating method.

LBL coating step	Sample code	Zeta‐potential, mV
0	VGCF	−41.8 ± 6.4
1	L1@VGCF	+39.7 ± 5.2
2	L2@VGCF	−51.5 ± 6.4
**3**	L3@VGCF	+38.5 ± 7.0
4	L4@VGCF	−45.5 ± 4.2
5	L5@VGCF	+36.6 ± 6.5
6	L6@VGCF	−46.7 ± 5.1

The polymer coating is further quantified by TGA‐MS as shown in Table S1. The weight percentages of polymer in L1@VGCF, L2@VGCF, and L6@VGCF are 2, 11, and 47 wt% (Figure S1), equivalent to the weight loss through TGA to 530°C. The thermal stability of L1@VGCF, L2@VGCF, and L6@VGCF is lower than VGCF (*T*_d_ = 725 °C, CO_2_, NO_2_ evolution), due to the lower decomposition temperature of the polymer. The presence of PDADMAC in L2@VGCF is characterized by simultaneous NO_2_ and CO_2_ evolution starting from 439 °C in the MS response. The PSS coating is characterized in the MS response by SO_2_ evolution starting at 305 °C resulting from decomposition of the sulfonate group [34]. A similar decomposition profile is observed for L6@VGCF (Figure S2, decomposition products starting from 410°C are CO_2_, NO_2_ indicating PDADMAC, and decomposition product SO_2_ from 319°C indicating PSS).

**FIGURE 2:**
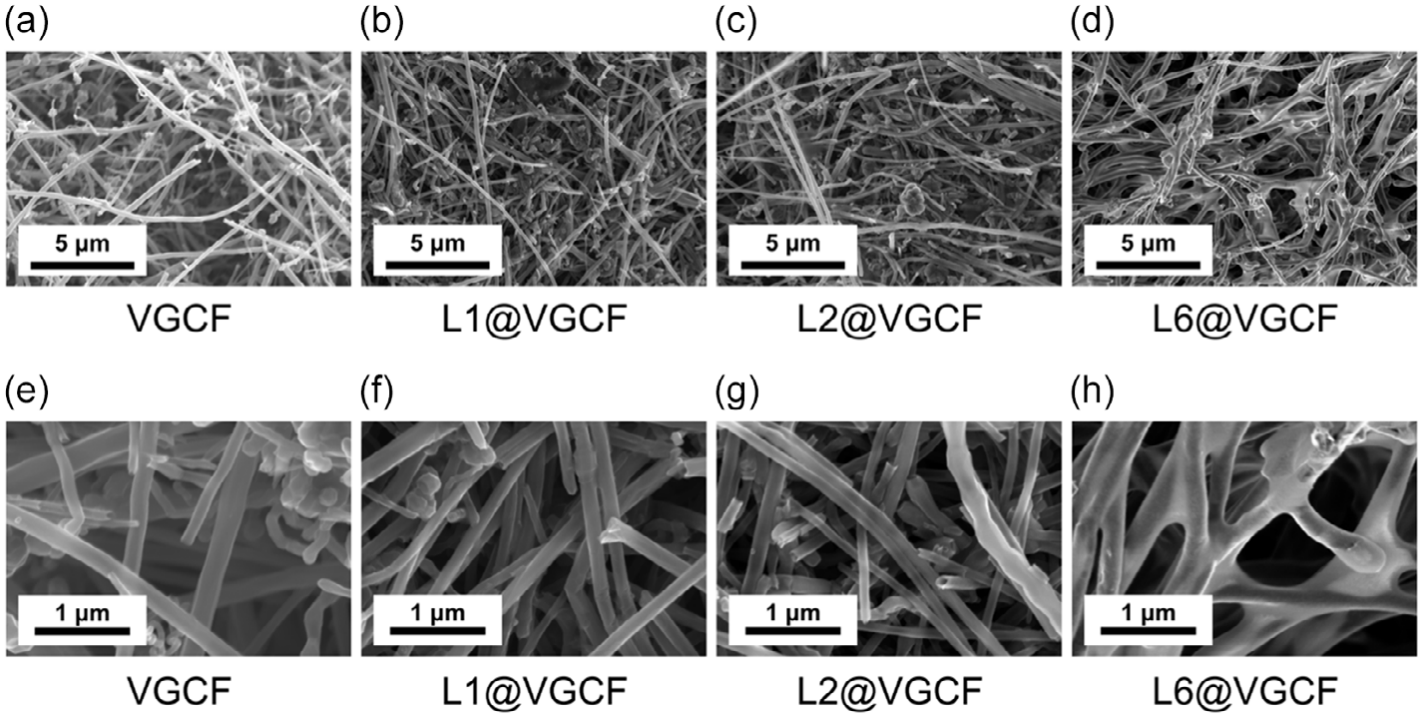
SEM secondary electron images (In‐Lens) of (a) VGCF, (b) L1@VGCF, (c) L2@VGCF, (d) L6@VGCF at magnification of 6000, and (e–h) the respective images at magnification of 30,000.

The morphologies of pristine and coated VGCF are analyzed by SEM. Figure 2 shows SEM images of pristine VGCF, L2@VGCF, and L6@VGCF. Polymer layers are evident for all coated samples. For L6@VGCF, an extended polymer network is observed, while the fibers appear to still be separated for L1@VGCF and L2@VGCF. Too high polymer layer thickness leads to aggregation of VGCF, forming a porous network, as the polymer acts more like a binder and fibers increasingly stick together.

To confirm a successful polymer coating, the average fiber diameters before and after polymer coating are compared. Furthermore, with these SEM images, it is possible to conclude whether the polymer coating ensues in a conformal manner. Ongoing from L1@VGCF to L6@VGCF, the average diameter of the coated fibers increases from 105 to 297 nm. The distinction is clear between very low and very high number of polymer layers. For VGCF and single‐layer samples, the thickness shows a distinct margin of error of the measurement. Nevertheless, the coating thickness evidently increases with increasing number of deposition steps.

XPS surface analysis of L6@VGCF further confirms the presence of polymer at the surface of VGCF, supporting TGA‐MS and SEM. The XPS spectrum and data are shown in Figure S3 and Table S2. The detection of photoelectrons with the characteristic binding energies for sulfur (S2p: 168.4 and S2s: 232.1 eV), oxygen/Na (O1s/NaKLL: 531.6 eV), chlorine (Cl 2p: 200.4 eV), and sodium (Na1s: 1071.6 eV) (from PSSNa polymer) proves a successful coating with PSS. Expected nitrogen from PDADMAC is not detected, probably due to the surface sensitivity of XPS. This elemental analysis proves a successful deposition of the PSS as the outermost layer.

**FIGURE 3:**
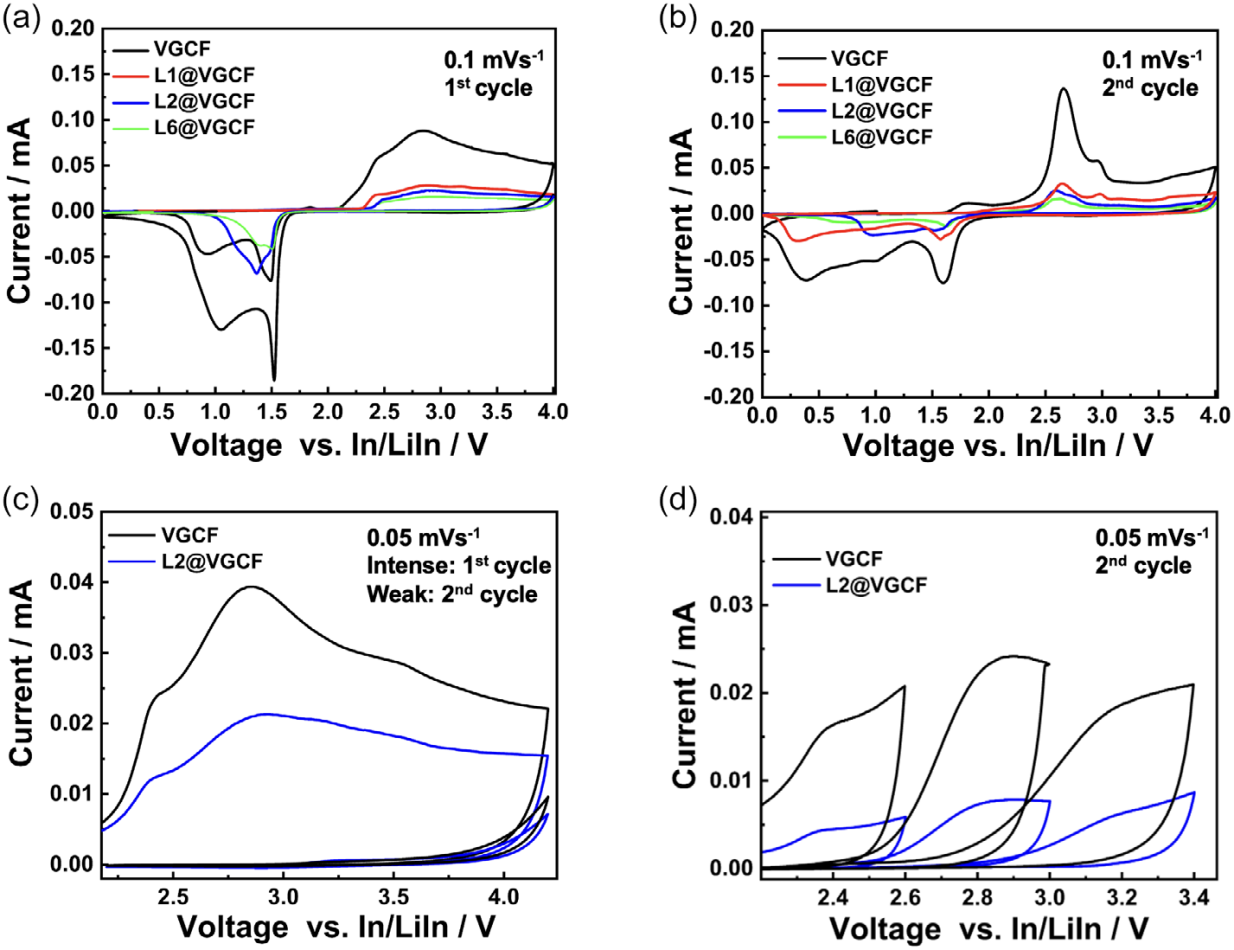
(a) CV of first cycle of LiIn|LPSCl|L*n*@VGCF‐LPSCl (*n* = 0; 1; 2; 6) cells at the scan rate of 0.1 mV·s^−1^. (b) CV of second cycle of LiIn|LPSCl|L*n*@VGCF‐LPSCl (*n* = 0; 1; 2; 6) cells at the scan rate of 0.1 mV·s^−1^. (c) CV for LiIn|LPSCl|L*n*@VGCF‐LPSCl (*n* = 0; 2) recorded at 0.05 mV·s^−1^ scan rate with narrow voltage range of 2.2–4.2 V at first and second cycles. (d) CV for LiIn|LPSCl|L*n*@VGCF‐LPSCl (*n* = 0; 2) scanned in stepwise manner between 2.6 and 3.4 V with interval of 0.4 V at 0.05 mV·s^−1^ scan rate.

To detect electrolyte oxidation only at the VGCF surface, we prepare LiIn|LPSCl|LPSCl‐VGCF cells, with either pristine VGCF or coated VGCF (L1@VGCF, L2@VGCF, and L6@VGCF). No cathode active material is added as electrolyte degradation of LPSCl at the LPSCl‐VGCF interface is studied using CV. The respective peak currents correspond to argyrodite oxidative and reductive decomposition and are used as parameters for the study. Figure 3a shows the CV data of the first cycle with oxidative and reductive segments for the respective cells. During oxidation, the LPSCl first forms LiCl and Li_3_PS_4_, followed by S and P_2_S_5_ upon further oxidation [35]. The oxidation peak at the onset voltage of 2.64 V is ascribed to the oxidation of S_*x*_^2−^ to S_*x*_ [35]. The second peak at 2.93 V is related to the formation of from the oxidation of PS_4_^3−^ to P_2_S_5_ [35]. The current response for the first oxidation segment is lowered to only 35.7% for L6@VGCF and 28.6% for L2@VGCF as compared to pristine VGCF (Table S3). The oxidation current (*I*_ox_ in mA) is used as the parameter for comparison. The weight of VGCF excluding polymer weight is considered for comparing the current response between the samples (*I*_ox_ in mA·mg^−1^).

In the second cycle (Figure 3b), a strong oxidation peak at 2.64 V occurs in all three cyclic voltammograms. Overall, the peak current response for the oxidation peaks is lower for L2@VGCF and L6@VGCF in both oxidation and reduction cycles than for pristine VGCF. In reduction cycles, a strong peak appears at 1.59 V. The peak at 1.59 V is assigned to the reduction of P_2_S_5_ and other polysulfide species (S_*x*_) to S_*x*_^2−^ [35]. The polymer coating does not alter the electronic properties of VGCF. Thus, the current response is influenced mainly by two factors: firstly, the coating on the VGCF surface, which mitigates oxidation of the SE; secondly, the aggregation of VGCF at higher coating thickness, as seen in SEM, which hinders homogeneous distribution of fibers inside the cathode composite, suggesting a lower contact area between VGCF and LPSCl. This leads to a decrease in the effective electrochemically active area due to partial electronic isolation. To enable a fair comparison between pristine and polymer‐coated samples, we did not take into account the weight of the polymer in such analysis in Table S3. All the reductions of current in percentage are evaluated as per only the VGCF weight.

Argyrodite is chemically unstable to polar solvents or polymers with polar functional groups. It is important to evaluate oxidative stability of polymer layers with argyrodite prior to battery application. The cyclic voltammogram in the second cycle for VGCF and polyelectrolyte‐coated VGCF (Figure 3b) did not exhibit any additional peaks. To further evaluate polymer stability and to exclude reduction of oxidized products, which evaluate subsequent electrolyte or polymer oxidation after the first cycle difficult, CV measurements of L2@VGCF and VGCF were conducted in a voltage range of 2–4 V and lower scan rate (0.05 mV·s^−1^). The voltage range of 2–4 V does not have any additional oxidation peaks related to polymer oxidation (Figure 3c).

The CV of the polyelectrolyte‐coated VGCF and bare VGCF is repeated stepwise from 2.6 to 3.4 V with 0.4 V at each step [35]. The experiment is designed to evaluate current response at each voltage step to deduce the electrolyte oxidation potential more accurately. This method can detect slower kinetics related to polymer decomposition, if present. Figure 3d presents second cycles recorded at each step. No further additional polymer oxidation peaks or sudden increase in current is observed. These combined experiments indicate that the polyelectrolyte coating is electrochemically and chemically stable with argyrodite.

To determine whether the prepared coatings influence SEB performance, L*n*@VGCF are compared in cells of LiIn|LPSCl|LPSCl‐L*n*@VGCF‐NCM configuration with *n* = 0; 1; 2; 6. Herein, the cathode composite consists of NCM cathode active material (69.3 wt%), LPSCl SE (29.7 wt%) for ionic percolation, and VGCF carbon additive (1 wt%) for electronic contact. Inside the composite, LPSCl is oxidized at NCM|SE and VGCF|SE interfaces during cycling. The polymer coating on VGCF is tested here as protective barrier toward electrolyte oxidation at the surface of the carbon additive. The electrochemical performance of polyelectrolyte‐coated VGCF is evaluated by galvanostatic charge–discharge cycling with LiIn|LPSCl|L*n*@VGCF‐LPSCl‐NCM (*n* = 0; 1; 2; 6) cells. The cells are cycled for one to five cycles with different VGCF.

**FIGURE 4:**
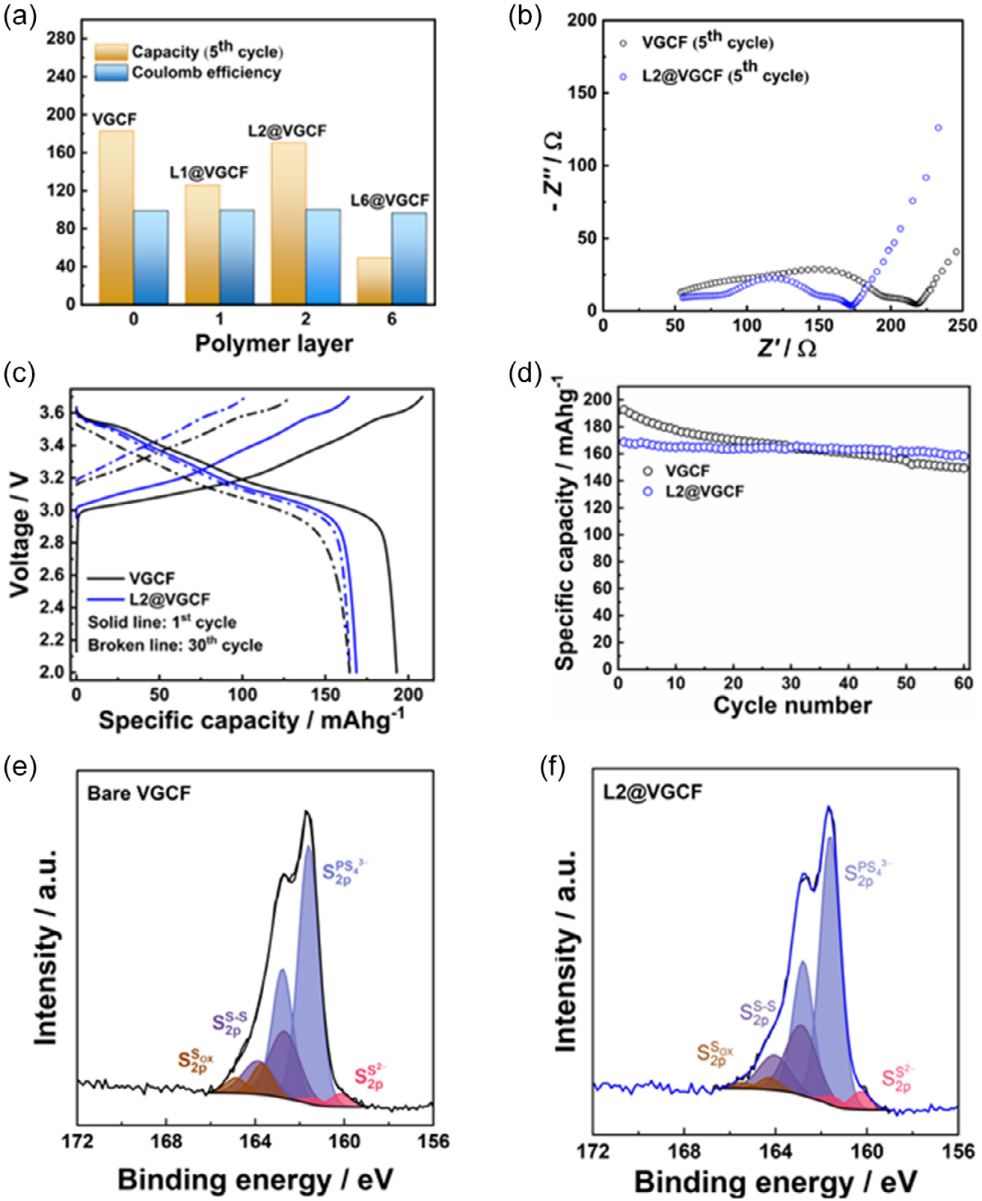
(a) Layer‐dependent cycling performance of LiIn|LPSCl|L*n*@VGCF‐LPSCl‐NCM (^LiIn^SEB^NCM^) cells (*n* = 0; 1; 2; 6), recorded at 0.1C rate for five cycles. (b) Nyquist plots for ^LiIn^SEB^NCM^ cells using coated and uncoated VGCF recorded at 3.14 V during fifth charge cycle. (c) Voltage profiles for ^LiIn^SEB^NCM^ cells using coated and uncoated VGCF for 1^st^ and 30^th^ cycles at 0.1C rate. (d) Cycling test of ^LiIn^SEB^NCM^ cells using coated and uncoated VGCF. (e) Ex situ XPS analysis of VGCF‐LPSCl‐NCM and (f) L2@VGCF‐LPSCl‐NCM cathode composites after 30 cycles at 0.1C rate.

Figure 4a presents the specific discharge capacities recorded after five cycles and CE for cells using the different L*n*@VGCF (*n* = 0; 1; 2; 6). The specific discharge capacity of NCM decreases with increase in polymer layers on VGCF from 183 mAh·g^−1^ for pristine VGCF to 50 mAh·g^−1^ for L6@VGCF. The CE with bare VGCF is 99.2% at fifth cycle and coating did not affect the CE for L1@VGCF (99.4%) and L2@VGCF (100%). For L6@VGCF, the CE is slightly less (96.6%). The decrease in capacity is correlated to the decrease in current response in CV. For L6@VGCF, the polymer forms an extended isolated network of VGCF, which may be hard to break while preparing the cathode composite, but it may also be related to VGCF aggregation due to the polymer coating. On the one hand, the coating decreases electrolyte oxidation, but on the other hand, the coating may lead to loss of the electronic conductive network when the coating is too thick. The L2@VGCF performs better than L1@VGCF, possibly due to the stability of the coating, owing to stronger electrostatic attraction between polycation and polyanion.

The corresponding EIS spectra recorded at first and fifth charge cycles for VGCF and L2@VGCF are shown in Figure S4 and Figure 4b, respectively. Before recording impedance, the battery is kept at rest at 3.15 V for 10 hr. In the first cycle, the overall cell resistance for L2@VGCF containing cells is 106 Ω, which is similar to that of cells with pristine VGCF (101 Ω). Initially, the polymer coating does not affect the resistance to a significant extent. Notably, in the fifth cycle, the overall cell resistance for L2@VGCF is lower (173 Ω) than that of uncoated VGCF (218 Ω). The polymer coating might improve the contact within cathode composites and decrease SE degradation. This explains the lower resistance of the cell with coated VGCF during cycling.

Longer cycling and ex situ analysis are performed using L2@VGCF and pristine VGCF. Figure 4c shows the voltage profile of a cathode composite using VGCF and L2@VGCF. The specific discharge capacity of VGCF decreases from 192.8 mAh·g^−1^ in the first cycle to 149.3 mAh·g^−1^ after the 60^th^ cycle (23% capacity loss). For the L2@VGCF sample, the specific capacity decreases from 168.7 mAh·g^−1^ in the first cycle to 158.1  mAh·g^−1^ after the 60^th^ cycle (6% loss) (Figure 4d). This demonstrates improved cycling stability with the coating as compared to the cells using pristine VGCF. Likely, this improvement is related to the lower internal resistance increase during cycling.

Figure 4e,f show the results of ex situ XPS performed on the composite cathode using VGCF and L2@VGCF, using a cell that was disassembled carefully after 30 cycles. The argyrodite decomposes at the current collector, VGCF, and cathode active material interfaces with the SE. As the decomposition products with the current collector are not the subject of study, depth profiling is carried out from the current collector side to investigate only the decomposition products deeper inside the composite cathode. Table S4 lists the detailed components as analyzed by XPS, showing in total four doublets for S2p bands. The main component at 161.6 eV is indicative of the PS_4_ unit of LPSCl electrolyte [18, 20]. The doublets at higher binding energies at 162.7 and 163.7 eV are due to polysulfides or oxidized (S–S and various S_Ox_) compounds [18, 36, 37]. The doublet at lower binding energy (160.1 eV) corresponds to Li_2_S [18, 36], which may originate from sulfide electrolyte synthesis. Both samples show similar decomposition products. No additional decomposition product is detected. It is not expected that the polymer‐coated VGCF can exhibit different decomposition products, as the polyelectrolyte complex is chemically stable with argyrodite. The atomic percentage of oxidation decomposition products (*S*_ox_) in coated VGCF (3.0 at%) is less compared to VGCF (9.1 at%), supporting the cycling data. Coating mitigates oxidation of argyrodite. As no additional peak is detectable, this indicates that the polymer coating is stable inside the cathode composite.

**FIGURE 5:**
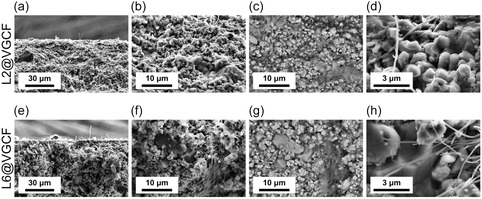
Cross‐sectional SEM images of composite cathodes for (a–d) L2@VGCF‐LPSCl‐NCM and (e–h) L6@VGCF‐LPSCl‐NCM, cycled at 10 cycles prior to analysis. The secondary electron detector is used for (a,b,d) and (e,f,h). The energy‐selective backscattered detector is used for (c,g).

Figure 5 shows a cross‐sectional view of cathode composites employing L6@VGCF‐LPSCl‐NCM and L2@VGCF‐LPSCl‐NCM after 0 cycles. This experiment is designed to investigate the reason behind the variation in capacity between L6@VGCF and L2@VGCF in terms of the morphology of polymer‐coated VGCF inside the cathode composite. The L6@VGCF sample shows significant VGCF aggregation. The aggregated region exists as islands throughout the scanned samples. In contrast, the VGCF aggregation is not prominent in SEM images for L2@VGCF‐LPSCl‐NCM. This confirms that higher polymer content leads to VGCF aggregation. Such aggregation of VGCF results in a significant lowering of specific capacity for L6@VGCF compared to L2@VGCF (Figure 5a). These results demonstrate how L2@VGCF is the best‐performing polyelectrolyte complex coating for improving the cycling stability of ^LiIn^SEB^NCM^.

## 4 Conclusion

We present a polyelectrolyte complex (PDADMAC:PSS) as coating for carbon fibers in argyrodite‐type SEBs. The electrostatic interaction between polycation and polyanion creates a stable coating on the carbon surface. CV is used to study the oxidation and reduction of LPSCl at the interface of VGCF and LPSCl. It confirms that the polyelectrolyte coating reduces oxidation of argyrodite while maintaining electrochemical stability of polymers inside the cathode composite. CV evaluates electrochemical stability of polyelectrolyte complex coating at the LPSCl and VGCF interface. SEM cross‐sectional images of cathode composites show that excessive polymer layer thickness causes VGCF aggregation, when more than two polymer layers are applied. Fine‐tuning of polymer content and coating thickness is mandatory to achieve improved cycling stability. The use of such polyelectrolyte complex interlayers for cathode and electrolyte surfaces is a promising approach for improving the cycling stability of solid‐state batteries.

## Supporting Information

Additional supporting information can be found online in the Supporting Information section. Supporting information includes figures and tables related to sample analysis and electrochemical testing. This material is available free of charge via the Internet at https://pubs.acs.org/.

## Author Contributions

S.S. carried out the electrochemical experiments, general materials characterization, and data analysis. B.‐X.S. contributed to the electrochemical experiments and the manuscript preparation. N.H. and F.S. contributed to materials preparation, process optimization, and cyclic voltammetry. F.W., J.S., and F.H.R. helped with the carried‐out characterization methods and data analysis. S.S. and F.H.R. conceived the idea and prepared the manuscript. All authors contributed to the manuscript and analysis of results.

## Funding

This study was supported by the German Federal Ministry of Research, Technology and Space (03XP0261).

## Conflicts of Interest

The authors declare no conflicts of interest.

## Supporting information

Supplementary Material

## Data Availability

The data that support the findings of this study are available from the corresponding author upon reasonable request.
