# Supplementary material for: Polyelectrolyte Complex Coating for Mitigating Decomposition at Argyrodite and Conductive Carbon Interfaces in Solid‐State Batteries
Source: ChemSusChem. 2026 Apr 18;19(8):e202502431. doi: 10.1002/cssc.202502431 (PMC13091074; doi:10.1002/cssc.202502431)
Supplement: Supplementary file 1 — Supplementary Material [file CSSC-19-e202502431-s001.pdf]

Supporting information

Polyelectrolyte Complex Coating for Mitigating Decomposition at Argyrodite and Conductive Carbon Interfaces in Solid-State Batteries

Sudeshna Sen[1,2], Bing-Xuan Shi[1,2], Nina Herrmann[1,3], Felix Schnaubelt[1,2], Felix Walther[1,4], Joachim Sann[1,2], Felix H. Richter[1,2,*]

[1]Institute of Physical Chemistry, Justus-Liebig-University Giessen, Heinrich-Buff-Ring 17, 35392 Giessen, Germany

[2]Center for Materials Research, Justus-Liebig-University Giessen, Heinrich-Buff-Ring 16, 35392 Giessen, Germany

[3]Present address: VSPC Pty Ltd, Brisbane, Queensland, Australia.

[4]Present address: ZEISS Semiconductor Manufacturing Technology, Oberkochen, Germany.

*Corresponding author: felix.h.richter@phys.chemie.uni-giessen.de

Table S1. TGA-MS and SEM results summarizing thermal decomposition in synthetic air and fiber diameter, respectively.

	**Weight loss of TGA at 530 °C / wt%**	**Decomposition temperature T_d / °C**	**Main gaseous decomposition products**	**Average fiber diameter / nm**
**VGCF**	0	725	CO_2, NO_2	126 ± 40
**L1@VGCF**	2	214	SO_2	105 ± 32
		635	CO_2, NO_2	
**L2@VGCF**	11	305	SO_2	117 ± 32
		410	SO_2	
		439	CO_2, NO_2	
		598	CO_2, NO_2	
**L6@VGCF**	47	319	SO_2	297 ± 90
		410	CO_2, NO_2	
		556	CO_2, NO_2	

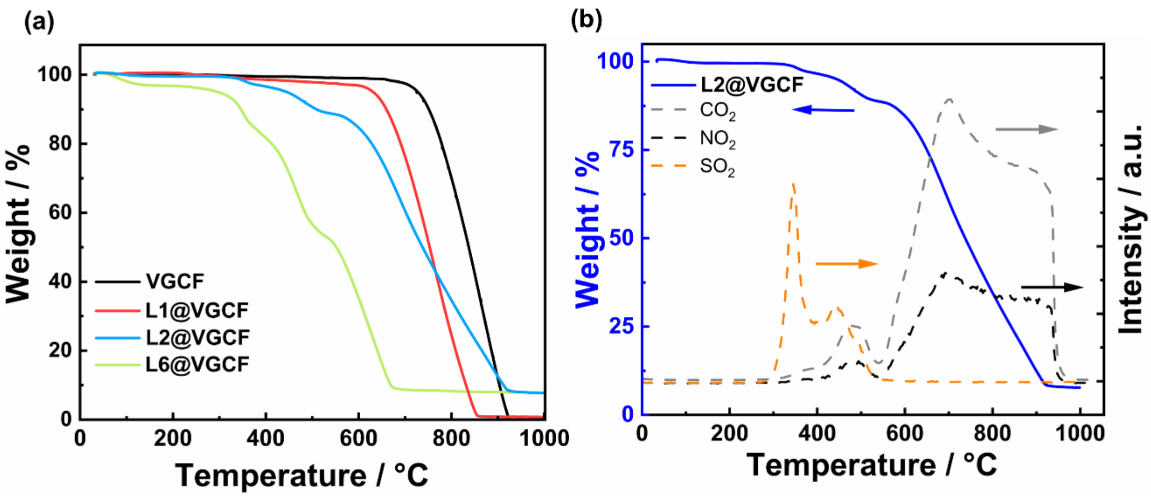

Figure S1. (a) TGA of L*n*@VGCF (*n* = 0; 1; 2; 6) recorded at 10 K min^{-1} in synthetic air. (b) TGA-MS of L2@VGCF recorded at 5 K min^{-1} in synthetic air. The intensity of NO_2 and SO_2 is multiplied by 100.

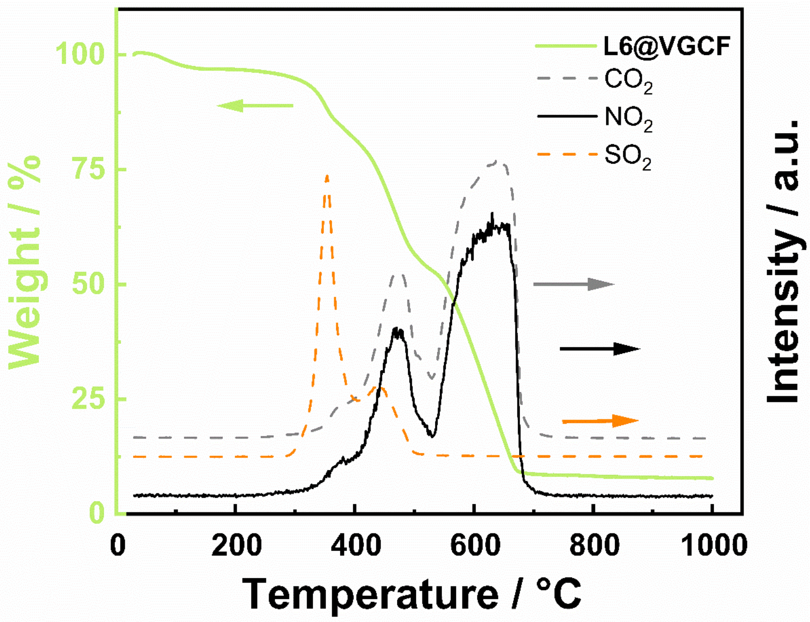

Figure S2. TGA coupled mass spectrometry analysis of L6@VGCF recorded at 10 K min^{-1} in synthetic air.

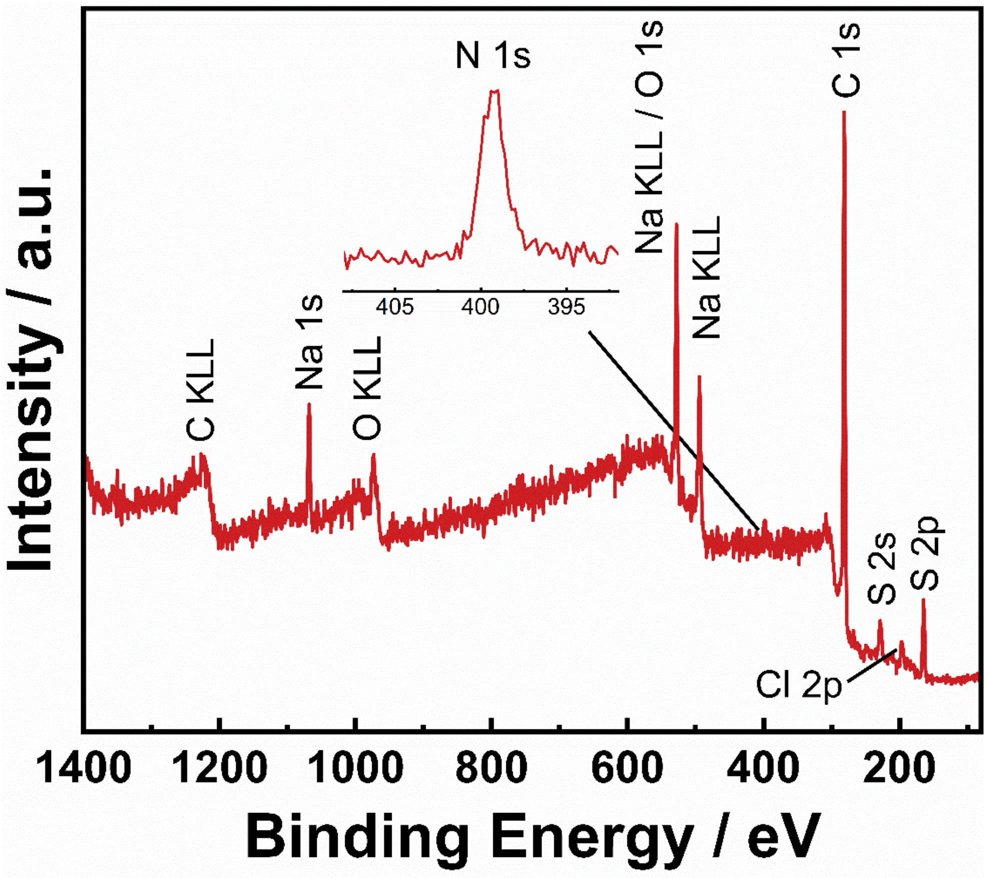

Figure S3. XPS surface analysis of L6@VGCF coated sample.

Table S2. The binding energy of each element obtained from XPS analysis of L6@VGCF, characterizing the PSS structure on surface of VGCF.

Sample L6@VGCF	E_{bind} / eV
S2p	168.4
S2s	232.1
Cl2p	200.4
C1s	284.8
N1s	402.6
O1s / Na KLL	531.6
Na1s	1071.6

Table S3. Cyclic voltammetry results of L*n*@VGCF (*n* = 0; 1; 2; 6) and specific capacity obtained from galvanostatic charge discharge cycling for LiIn|LPSCl|L*n*@VGCF-LPSCl-NCM. The peak current response for first oxidation segment at 2.65 V is denoted as I_{ox}.

Sample	I_{ox} / mA	m_{VGCF} / mg	I_{ox} / mA mg^{-1} of VGCF	I_{ox} / %	q at 5th cycle / mAh g^{-1} of NCM
**VGCF**	0.087	3.03	0.028	-	183
**L1@VGCF**	0.028	2.73	0.010	35.7	126
**L2@VGCF**	0.022	2.73	0.008	28.6	170
**L6@VGCF**	0.016	1.52	0.010	35.7	49

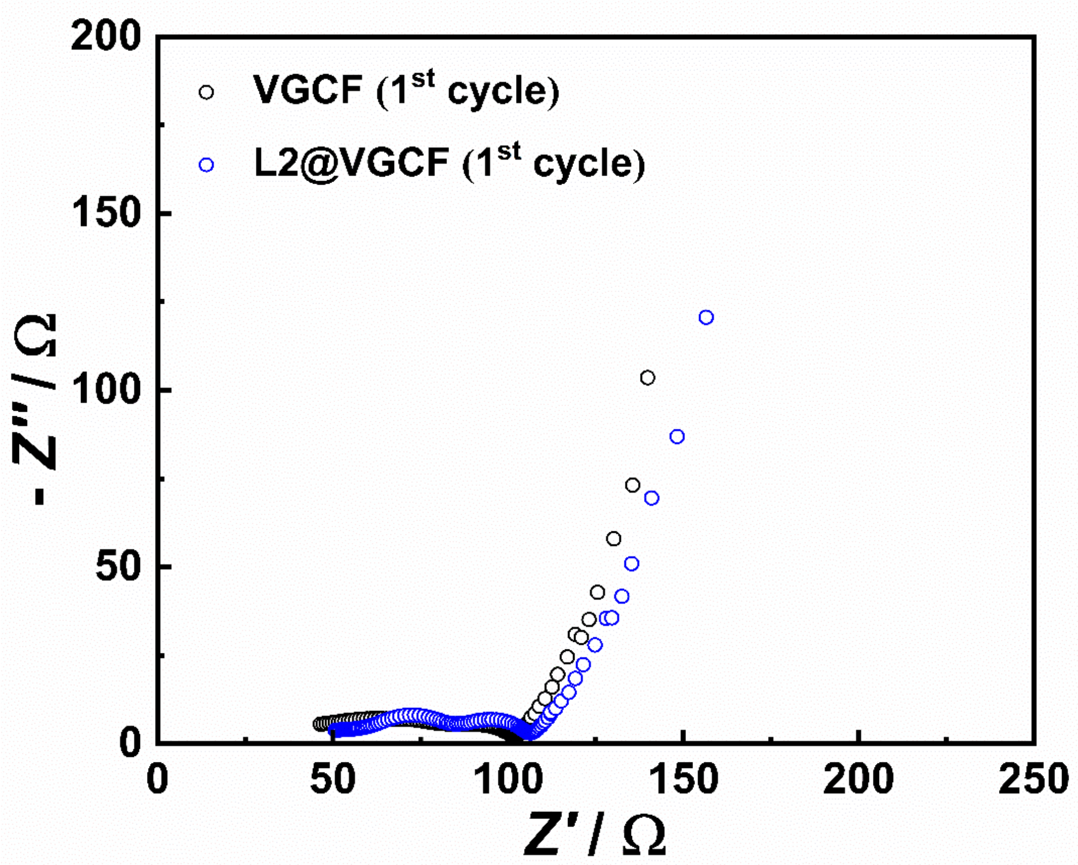

Figure S4. Nyquist plot for LiIn|LPSCl|L*n*@VGCF-LPSCl-NCM cell (*n* = 0; 2) recorded at 3.14 V during the first charge cycle.

Table S4. The atomic percentage and binding energy of decomposition products as obtained from ex-situ XPS analysis.

**Atomic percentage of**	**S^{2-} / at%**	**PS_4 / at%**	**S-S / at%**	**S_{Ox} / at%**
**VGCF**	3.8	58.0	29.1	9.1
**L2@VGCF**	4.8	66.2	26.4	3.0
**The binding energy of**	**S^{2-} / eV**	**PS_4 / eV**	**S-S / eV**	**S_{Ox} / eV**
**VGCF**	160.1	161.6	162.7	163.7
**L2@VGCF**	160.2	161.6	162.9	164.3
